# Mutational Analysis of Cvab, an ABC Transporter Involved in the Secretion of Active Colicin V

**DOI:** 10.1371/journal.pone.0035382

**Published:** 2012-04-23

**Authors:** Kai-Hui Wu, Ying-Hsin Hsieh, Phang C. Tai

**Affiliations:** Department of Biology, Georgia State University, Atlanta, Georgia, United States of America; Institut Pasteur Paris, France

## Abstract

CvaB is the central membrane transporter of the colicin V secretion system that belongs to an ATP-binding cassette superfamily. Previous data showed that the N-terminal and C-terminal domains of CvaB are essential for the function of CvaB. N-terminal domain of CvaB possesses Ca^2+^-dependent cysteine proteolytic activity, and two critical residues, Cys32 and His105, have been identified. In this study, we also identify Asp121 as being the third residue of the putative catalytic triad within the active site of the enzyme. The Asp121 mutants lose both their colicin V secretion activity and N-terminal proteolytic activity. The adjacent residue Pro122 also appears to play a critical role in the colicin V secretion. However, the reversal of the two residues D121P - P122D results in loss of activity. Based on molecular modeling and protein sequence alignment, several residues adjacent to the critical residues, Cys32 and His105, were also examined and characterized. Site-directed mutagenesis of Trp101, Asp102, Val108, Leu76, Gly77, and Gln26 indicate that the neighboring residues around the catalytic triad affect colicin V secretion. Several mutated CvaB proteins with defective secretion were also tested, including Asp121 and Pro122, and were found to be structurally stable. These results indicate that the residues surrounding the identified catalytic triad are functionally involved in the secretion of biologically active colicin V.

## Introduction

CvaB is the central membrane transporter of the bacteriocin colicin V secretion system in *Escherichia coli*. It has been shown that CvaB, together with CvaA and TolC proteins, directs the secretion of the colicin V with the cleavage of a double-glycine leader peptide [1], [2], [3]. Sequence alignments indicate that CvaB protein belongs to the ABC superfamily [2]. It is predicted to span the membrane six times and contains an ATP-binding site in its C-terminal cytoplasmic domain [2], [4]. The amino acid sequence of this ATP-binding domain is highly conserved among many proteins involved in export processes, including the mammalian MDR family of drug exporters [5], [6] and CFTR, the chloride channel defective in patients with cystic fibrosis [7], [8]. For the export of bacteriocin precursors containing a short leader peptides of the double-glycine type, such as colicin V, it has been proposed that the substrate-binding site in the proteolytic domain binds a bacteriocin precursor, and hydrolysis of ATP molecule(s) induces a conformational change in the ABC transporter that results in the simultaneous cleavage and translocation of the peptide [9]. Point mutations in the CvaB N-terminal putative protease domain and C-terminal nucleotide-binding motif severely affect colicin V secretion, suggesting that these two domains are critical for CvaB function [4], [10].

Previous work [10] has shown that the N-terminal domain of CvaB (BntD) possesses Ca^2+^-dependent cysteine proteolytic activity. Mutations on critical residues Cys32 and His105 totally abolish its proteolytic activity and colicin V secretion, indicating that the BntD is indeed important in colicin V precursor processing. Based on protein sequence analysis, the BntD belongs to the C39 peptidase superfamily, which contains the Cys-His-Asp sequence as a catalytic triad. Furthermore, Dessens *et al.* [11] reported that Asp forms hydrogen bonds with His to help orient the imidazole ring of the His to lie in a favorable position to interact with the Cys. In this work sequence alignment and site-directed mutagenesis indicate that Asp121 forms part of the catalytic triad and is important for colicin V secretion. Molecular modeling and sequence alignment of the N-terminal domain of CvaB have been used to predict its 3-D protein structure and locate potentially important residues in the vicinity of active sites of this cysteine protease. Pro122, Gln26, Trp101, Asp102, Val108, Leu76, and Gly77 in the neighborhood of the critical Cys32, His105 and Asp121 are shown to be functionally important in the secretion of a biologically active colicin V.

## Results

### Identification of Asp121 as the third residue in the CvaB proteolytic catalytic triad

**Figure 1:**
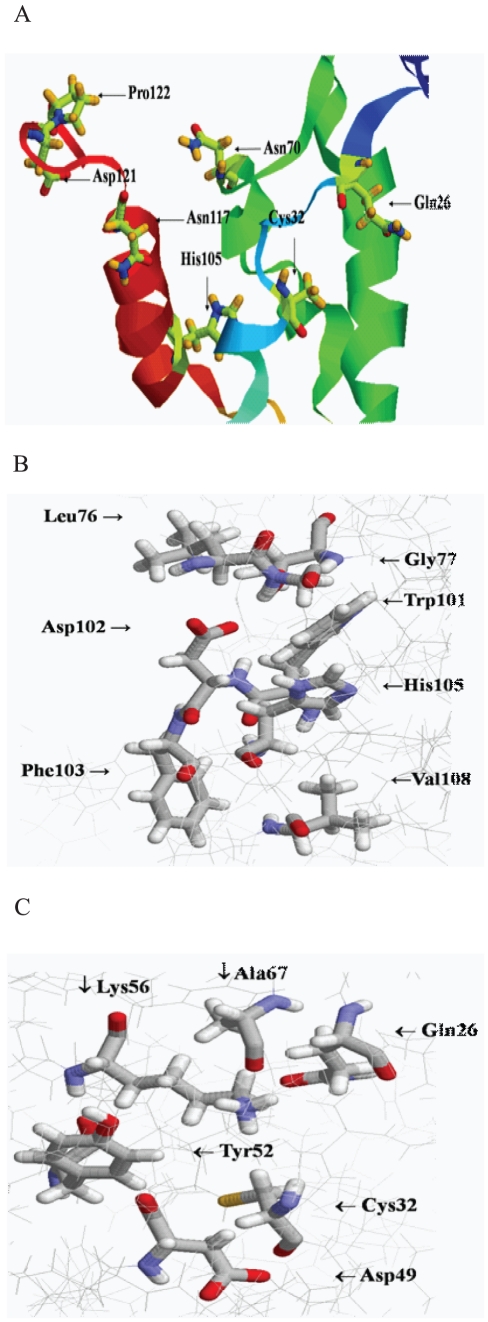
Molecular modeling of CvaB N-terminal domain in the vicinity of Cys32 and His105 residues. Coordinates of the crystal structures of the *E.coli* translation inhibitor (PDB: 1JD1) and photosynthesis metal transporter (PDB: 1G8P) were obtained from protein database (www.rcsb.org/pdb) and used as homolog templates for the molecular modeling. Amino acid residues 1 through 87 are based on template 1G8P, and residues 88 through 125 are based on template 1JD1. (A) The catalytic triad. Asn70 (7.68 Å), and Asn115 (14.9 Å) are the only two Asn residues close to His105. Asp121 (18.52 Å to His105) is the third residue for the catalytic triad. Gln26 is 7.6 Å to Cys32. (B) Trp101, Asp102, and Val108 are close to His105 to form potential H-bonds (4.15 Å for Trp101, 3.2 Å for Asp102 and 3.4 Å for Val108). Three residues, Leu76, Gly77, and Thr80, around Trp101 and Asp102 are also shown. (C) The critical residue Cys32 is shown with the surrounding residues, Gln26 (7.6 Å), Asp49 (3.83 Å), Tyr52 (3.4 Å), Lys56 (4.99 Å), and Ala67 (5.11 Å ).

**Figure 2:**
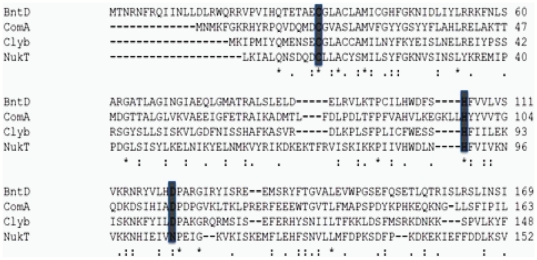
Alignment of N-terminal CvaB (BntD) with the C39 peptidase family. Amino acid multiple sequence alignments were performed using the ClustalW2 program. The sequences were taken from the Swiss Protein Database or the GenBank™/EBI Data Bank. Identical and similar residues are marked with *asterisks* and *dots*, respectively. The three conserved catalytic triad residues are marked as dark gray color. The *numbers* on the left and right refer to amino acid positions. The BntD is the N-terminal protease domain of CvaB (a.a. 1–171), ComA protein is a bacteriocin-processing peptidase from *Streptococcus pneumonia*, Clyb protein is a mersacidin lantibiotic processing peptidase from *Streptococcus pneumonia*, NukT is family C39 non-peptidase homologues from *Staphylococcus warneri*.

Previous studies [10] showed that Cys32 and His105 are critical residues for cysteine proteolytic activity of the N-terminal domain of CvaB and for colicin V secretion activity. We therefore searched for the third residue of the catalytic triad. It has been suggested that either Asn or Asp residues can complete the catalytic triad, working along with the Cys and His residues to give proteolytic activity [11]. It has also been suggested that either Asn or Asp are involved in hydrogen bonding with the requisite, active site His residue to stabilize the thiolate-imidazolium ion pair [12], [13]. The molecular modeling of N-terminal of CvaB (BntD) showed that Asn70 and Asn115 are the only two Asn residues close to His105 (Fig. 1A), though neither lies within the conserved region comparable to the C39 protease family sequence alignment (Fig. 2). Nevertheless, site-directed mutagenesis on these two residues changing them with Gln and Lys, respectively were carried out. The mutations, however, did not affect colicin V secretion (Table 1), indicating that neither Asn70 nor Asn115 is involved in the catalytic triad of proteolytic activity or secretion. According to sequence alignment of the catalytic triad (Fig. 2), Asp121 could be the third conserved residue of N-terminal CvaB and be involved in catalytic triad formation. Accordingly, this residue was modified using site-directed mutagenesis. Replacements of Asp121 with Ala, Ser and Asn lost all colicin V secretion activity (Table 1). All the mutants were tested at 30°C, 37°C, and 42°C, and none of them showed secretion activity, indicating that Asp121 is indeed essential for colicin V secretion activity. Surprisingly, mutant D121E showed partial secretion activity (about 25%) at 30°C and 37°C, but not 42°C, indicating that the carboxyl group of Asp might be important for colicin V secretion.

**Table 1:** The Mutation of Conserved residues on colicin V secretion.

Residue	Mutations	Activity
Asn70	N70G, N70K	++++
Asn115	N115G, N115K	++++
Asp121	D121A, D121S, D121N	_
	D121E	+
Pro122	P122A, P122S, P122G, P122F, P122V	_
Asp121/Pro122	D121P-P122D	_

Mutation and enzymatic activity are defined as in Material and Methods.

### The importance of Asp121/Pro122 for proteolytic activity

Pro122 is a conserved residue in the N-terminal CvaB neighboring, as it does, Asp121. Mutations of Pro122, including changing it to an Ala (P122A) and a Ser (P122S) resulted in defective colicin V secretion at 30°C, 37°C, or 42°C (Table 1). Based on the 3D model prediction (Fig. 1A), Pro122 residue is located at the alpha-helix turn that may be important for the maintenance of the helix structure. Consequently we mutated the Pro122 further, changing it into Gly, Phe, and Val; no colicin V activity was found (Table 1). The reversal of both residues, in a double mutation of D122P/P122D, also resulted in the loss of any secretion activity (Table 1), showing the critical positions of these two amino acids in the secretion of colicin V. These results indicate that the residues Asp121/Pro122 and their alignments are important for the secretion of active colicin V.

**Figure 3:**
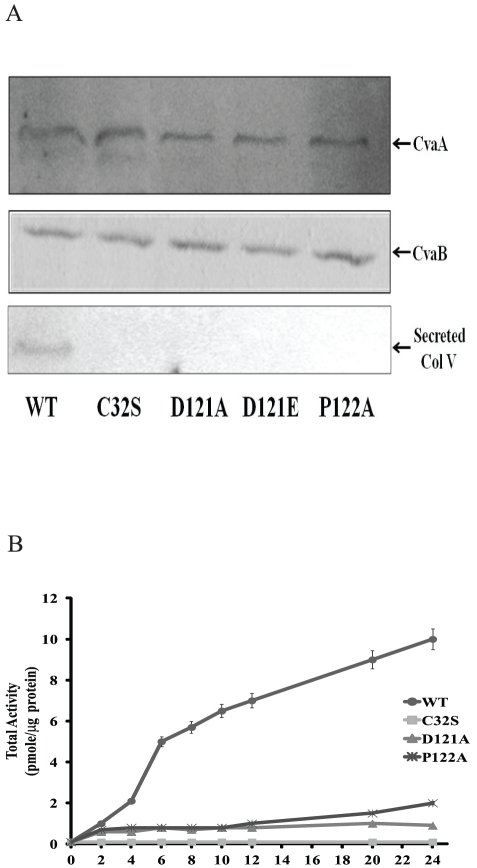
Protease stability and protein activity of Asp121 and Pro122. (A) Membranes of wild-type and mutants were analyzed by SDS-gel electrophoresis for immunoblots. The CvaB and CvaA were detected by CvaB and CvaA antibodies. The secreted colicin V from assay medium was precipitated by TCA and western blot with colicin V peptide antibody. (B).Purified BntD proteins of wild-type and mutants were incubated with LA-pNA with protease reaction buffer. The absorbance at 405 nm was monitored at the time indicated.

In order to determine whether the loss of colicin V secretion activity is due to protein degradation, we examined the stability of the mutated CvaB, as well as the stability of its partner CvaA. Such analysis, using immunoblots demonstrated that the CvaB and CvaA of Asp121 and Pro122 mutants are structurally stable (Fig. 3A), indicating that the loss of secretion activity is not due to the protein degradation of either CvaB or its associate protein, CvaA. In addition, we also determined the colicin V synthesis and secretion in the CvaB mutants. The amounts of colicin V synthesis in the cell were not affected in both wild type and mutants CvaB as expected (data not shown). However, immunoblot analysis of secreted colicinV indicated that the secretion was defective in the CvaB mutants (Fig. 3A, lower panel). These data confirmed that the loss of secretion activity of colicin V secretion is due to the defect of the N-terminal CvaB. The N-terminal CvaB mutant proteolytic domains, BntD-D121A and BntD-P122A, were cloned and purified to determine their proteolytic activity. Using LA-pNA as a substrate, the protease activity of both mutants was greatly reduced as compared to wild-type (Fig. 3B), with little protease activity being evident, even after 24 hours incubation. These data indicate that Asp121 and Pro122 are both critical for N-terminal CvaB protease activity and colicin V secretion.

### Additional important residues surrounding His105

Based on sequence alignment and protein modeling, there are several possible residues surrounding Asp121 which may also form H-bond interactions with His105 imidazole ring including Trp101, Asp102, Val108, Leu76 and Gly77. The mutations of these residues were characterized for their involvement in colicin V secretion activity.

### Trp101

It has been reported that Trp is proximal to His and Cys in the active site of *Conidiobolus* alkaline protease [14]. Molecular modeling of N-terminal CvaB predicts that Trp101 is 4.15 Å away from the active site His105. Replacement of Trp101 with Phe and Tyr showed retention of 75% of wild-type colicin V secretion activity, while 50% of colicin V activity was observed when Trp101 was replaced with Ala or Ser. Replacement of Trp101 with Asp (W101D) showed even less secretion activity (only 25%). However, replacement with His, Pro, and Lys showed no secretion activity at all (Table 2). These results strongly suggest that Trp101 plays an important role in colicin V activity, and that phenyl group of phenylalanine or tyrosine can effectively substitute for the indole ring of Trp101, potentially stabilizing the His105 residue during the catalytic reaction.

**Table 2:** Mutation of the residues around the His 105 on colicin V secretion.

Residue	Mutations	Activity
Trp101	W101Y, W101F	+++
	W101A, W101S	++
	W101D	+
	W101K, W101P, W101H	−
Asp102	D102K, D102G,D102Y, D102H, D102T, D102N, D102Q	++++
	D102S, D102E, D102C	+++
	D102W	+
	D102A, D102F, D102P,	−
	W101D -D102W	−
Val108	V108L	+++
	V108S	++
	V108A	+
	V108K, V108D	−

Mutation and enzymatic activity are defined as in Material and Methods.

### Asp102

It has been suggested that an Asp residue is involved in hydrogen bonding network to the catalytically critical His residue in cysteine protease. In so doing, it is thought to stabilize the active-site thiolate-imidazolium ion pair and enhance catalytic activity [15], [16]. Based on the molecular modeling prediction, the oxygen atom of Asp102 in CvaB is 3.2 Å away from the nitrogen of the His105 imidazole ring. To determine whether Asp102 is important in this function of N-terminal CvaB, colicin V activity assays were performed in selected mutants. Replacement of Asp102 with Lys, Tyr, Gly, Thr, Gln, Asn, and His showed comparable secretion activity to wild-type (Table 2). Replacement of Asp102 to Ser, Cys, and Glu showed 75% of wild-type colicin V secretion activity, while Ala, Phe, and Pro replacements completely abolished activity (Table 2). Moreover, double mutations of W101D & D102W totally abolished secretion activity, indicating that exchange of residues at position 101 and 102 cannot complement the amino acid property and the indole ring is important at the position of residue 101. Based on these mutagenesis results, hydrophilic residues appear to be required at the position 102.

### Phe103

Phe103 is another residue nearby Tyr101 which could also provide a phenyl ring in close proximity to His105. Even so, according to 3-D structure prediction, they are not considered to be neighbors. Replacement of Phe103 to Tyr or Trp showed 100% secretion activity comparable to wild-type; moreover, replacements to hydrophobic Ala, hydrophilic Ser, and charged His, did not change colicin V secretion activity, indicating that Phe103 is not important for CvaB function the secretion of active colicin V (Data not shown).

### Val108

According to the N-terminal CvaB molecular modeling, Val108 is 3.4 Å away from His105 residue. Replacement of Val108 to Leu (V108L) resulted in a slight reduction in the secretion activity (to 75%); replacement with Ser reduced activity further (to 50%); while replacement to Ala reduced activity even further (to 25%). Mutations of Val108 to charged residues Lys and Asp totally abolished secretion activity (Table 2). Interestingly, when the V108A mutation was combined with any other second mutation, the colicin V secretion activity was totally abolished (data not shown). These results indicate that Val108 is also important in the secretion of active colicin V.

### Trp101, Asp102, and Val108 mutants are structurally stable

**Figure 4:**
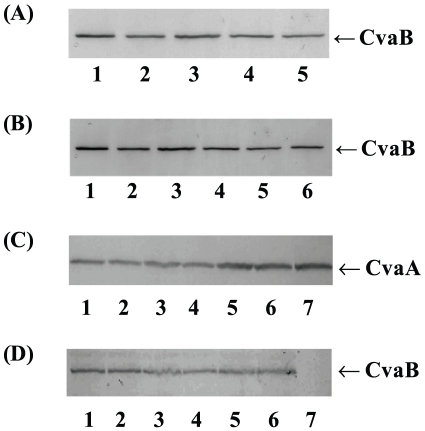
Stability of CvaA and CvaB in the membranes of Trp101, Asp102, and Val108 mutants. Wild-type and mutants *cvaB* plasmids were co-transformed with pHK11-4 plasmid lacking *cvaB* and incubated at 37°C. (A) Trp101 mutants CvaBs were detected by Flag antibodies. Lane 1, wild-type; 2, W101Y; 3, W101K; 4, W101P; 5, W101H mutants. (B) Asp102 mutant CvaBs were detected by Flag antibodies. Lane1, wild-type; lane 2, D102F; lane 3, D102A; lane 4, D102P; lane 5, D102W & W101D; lane 6, D102K mutants. Val108 mutant membranes were detected with CvaA antibodies (C) or by N-terminal domain of CvaB antibodies (D). Lane 1, wild-type; lane 2, V108L; lane 3, V108S; lane 4, V108A; lane 5, V108K; lane 6, V108D; lane 7, pHK11-4. The samples of membrane proteins were run on 10% SDS-PAGE and subjected to immunoblotting as described in Materials & Methods.

To determine whether the mutations that abolish secretion activity are due to functional or structural defects, the stability of CvaB, and its interacting partner, CvaA were determined. Flag-epitope was inserted into the C-terminal of CvaB wild-type, Trp101, and Asp102 mutants (SI Table 1) to detect CvaB; the insertions of Flag-epitope have no effect on the secretion of active colicin V. To determine whether the lost activity of several Trp101 mutants was due to functional defects or structural instability, membrane protein preparations from these mutants were electrophoresed on SDS-PAGE and subjected to immunoblot analysis. The results showed that the mutated CvaBs are structurally stable and can be detected immunologically the otherwise inactive W101Y, W101K, W101P, and W101H mutants as detected by Flag antibodies (Fig. 4A). Similar results were obtained with Asp102 mutants D102F, D102A, D102P, D102W&W101D, and D102K (Fig. 4B), suggesting that loss of activity in functionally compromised Asp102 mutants was due to functional defect.

Similarly, membrane proteins of V108A mutants were tested for CvaA and CvaB stability through immunoblotting. The results showed that CvaA (Fig. 4C) and CvaB proteins (Fig. 4D) of V108L, V108S, V108A, V108K, and V108D mutants could all be detected immunologically, indicating that secretion defect of these mutants are not due to structural instability of the mutated CvaB. We conclude that Trp101, Asp102 and Val108 are functionally involved in colicin V secretion, possibly through interactions with the critical residue, His105.

### Important residues around Trp101 and Asp102

Molecular modeling showed that Leu76, Gly77, and Thr80 residues are around important residues Trp101 and Asp102 (Fig. 1B). Leu76 is 2.9 Å to Asp102 and 3.1 Å to Thr80. Gly77 is 3.7 Å to Trp101, 4.3 Å to Asp102, and 4.3 Å to His105. Thr80 is 3.2 Å to both Trp101 and Asp102. To determine whether these three residues may be potentially involved in the secretion of active colicin V, site-directed mutagenesis was performed. Replacement of Gly77 for Ala or Ser reduced colicin V secretion activity by 50%; replacement of Gly77 to Pro totally abolished activity; replacements of Leu76 to Ala, Ser, and Val reduced activity to 50% at 37°C while replacement of Thr80 to Ser had no effect. Interestingly, replacements of Leu76 to Ala, Ser, or Val retained most colicin V activity at 30°C, but not at 42°C (Table 3 and data not shown). These results indicated that Leu76 and Gly77 are temperature-sensitive mutations affecting colicin V secretion activity.

**Table 3:** Colicin V secretion activity on the conserved residue, Leu76, Gly77 and Tyr80.

Mutations	37°C	42°C
CvaB WT	++++	++++
L76A	++	−
L76S	++	−
L76V	++	+
G77A, G77S	++	++
G77P	−	−
T80S	++++	++++

Mutation and enzymatic activity are defined as in Material and Methods.

### Important residues surrounding Cys32: involvement of Gln26

Previous studies showed that Cys32 is also critical for both colicin V secretion activity and cysteine proteolytic activity of N-terminal CvaB (BntD) [10]. Furthermore sequence alignment shows that Gln26, corresponding to Gln19 in papain, is conserved among the cysteine proteases. It has been proposed that Gln19 in papain forms an “oxyanion hole" with Cys25, and that this, in turn, stabilizes the Cys- and His- ion pair structure of calcium-free human m-calpain. 3-D structural analysis of papain also shows that the oxygen atom of Gln99, also conserved among cysteine protease [15], [17], [18], is at a distance of 7.73 Å from the sulfur of the critical Cys105 residue (PDB∶1KFU) [19]. 3-D modeling of BntD along with sequence alignment predicts that the oxygen of Gln26 also lies at a distance of 7.6 Å to the sulfur of Cys32, suggesting that Gln26 might be the amino acid involved in cysteine proteolytic activity of N-terminal CvaB (Fig. 1C and Fig. 2).

Site-directed mutagenesis was performed to determine whether Gln26 is involved in colicin V secretion activity. Replacement of Gln26 to Ala and Asn (Q26A and Q26N, respectively) had only minimal effect on colicin V secretion; while replacements of Gln26 for charged residues Lys, Glu, and His completely abolished colicin V secretion at 37°C (Table 4). However, replacement of Gln26 to Glu (Q26E) only reduced colicin V secretion by 50% at 30°C. These results showed that the Gln26 residue is involved in colicin V secretion activity.

**Table 4:** Direct colony assay for Gln26 site-directed mutants.

Mutation	30°C	37°C
CvaB WT	+++++	+++++
Q26A	++++	++++
Q26N	++++	++++
Q26E	++	+
Q26K	+/−	+
Q26H	+/−	+

Mutation and enzymatic activity are defined as in Material and Methods.

Molecular modeling also indicated that four residues, Asp49, Tyr52, Lys56, and Ala67, are in the vicinity of critical residue Cys32 at a distance of 3.83 Å, 3.4 Å, 4.99 Å, and 5.11 Å, respectively (Fig. 1C) [12], [13], [17], [20]. Site-directed mutagenesis on Ala67, Lys56, and Tyr52 residues did not affect colicin V secretion activity (data not shown). However, replacements of Asp49 to Glu and Ser reduced secretion activity to 75%; while replacements of Asp49 for Lys and Ala reduced activity to 50% -indicating that hydrophilic and acidic residues might also play some role in the secretion mechanism.

## Discussion

Previous study identified Cys32 and His105 of N-ternal CvaB (BntD) as being two of the three residues that are critical for both colicin V secretion activity as well as its cysteine proteolytic activity [10]. Most cysteine proteases contain Cys-His-Asn as their catalytic triad. However, it has has been reported that Asp, can replace Asn as the third residue, necessary to form the critical hydrogen bond with the imidazole ring of the histidine in order to stabilize its interaction with the cysteine residue, and thus contribute to the structural integrity of the active site [12], [13], [17], [20]. Molecular modeling of BntD shows that Asn70, lying at a distance of 7.68 Å form His105, and Asn115, at a distance of 14.9 Å, are the only two Asn residues that are potentially close enough to the critical histidine residue to have any interaction. Even so, these distances would not be close enough to form H-bonds. There is, however a conserved aspartate residue (Asp121) which through sequence alignment could be a candidate for the third position in the catalytic triad [21]. The directed replacement of this aspartate residue for either alanine or asparagine (D121A and D121N, respectively) results in a completely defective colicin V. Replacement of Asp121 with the common third catalytic residue Asn in cysteine protease, however, failed to restore the colicin V secretion activity, which suggests a role of the carboxyl side chain of the aspartate residue in colicin V secretion. Although Asp cannot be considered an essential catalytic residue in the cysteine protease [22], [23], [24], surprisingly, the mutant D121E protein loses most of its protease activity and is temperature-sensitive for secretion of active colicin V, indicating that Asp121 is functionally important in the protease activity of CvaB. In the D121N mutation, the change of an OH group for an NH_2_ group eliminates the negative charge in the van der Waals surface of the side chain atoms. Thus, it has been suggested that the carboxyl group on the Asp would be important for the catalytic activity across different pH ranges [25], with the protease activity of D121N being more active in a high pH environment [26]. Moreover, replacement of Asp with Glu (D121E) partially retains the colicin V secretion activity, suggesting that the carboxyl group is indeed important for the colicin V secretion. It was reported that Trp- Phe- and Pro- of cysteine protease stack together to help forming and stabilizing the catalytic triad [27]. Based on the 3D modeling, the Pro 122 locates on the end of helix structure next to Asp121. All the mutations of Pro122 lose colicin V secretion activity. Thus, these mutations may change the interaction with Trp and Phe to destabilize the catalytic triad. Interestingly, the reversal of the two residues, D121P/P122D, result in loss of activity, indicating the importance of amino acid residues alignments.

For cysteine protease, it has been suggested that the hydrogen bond between the side chain of His and Asn is buried in a hydrophobic region composed mainly of the side chains of residues Phe and Trp [28]. Moreover, a Trp indole ring interacts with a protonated His imidazole ring to increase the p*K*_a_ and stability of His [14], [28]. The Trp-His interaction shields the hydrogen bond of His-Asn from external solvent to facilitate different steps in the catalytic mechanism of the enzyme [12]. Previous studies indicated that Trp is essential for the active site of an alkaline serine protease [14], and that the imidazole ring is essential for the proteolysis [29]. Since Asn is not involved in colicin V secretion, Trp101 is the only Trp residue that could potentially interact with His105 in this way. Site-directed mutagenesis of Trp101 showed that Trp-to-Phe and Trp-to-Tyr substitutions can be tolerated for activity, but five-carbon ring His and Pro substitutions cannot. Thus, this His-Trp interaction in colicin V system is probably important for the Cys-His ion pair stability and the catalytic activity through the effects of increasing the p*K*_a_ of His.

The molecular modeling predicts the charged Asp102 may interact with the nitrogen of the imidazole ring in His105 in the distance of 3.24 Å to form a hydrogen-bonding network. Replacements of Asp102 with hydrophobic residues, such as Ala, Trp, and Phe, totally abolish colicin V secretion activity. On the other hand hydrophilic residue replacements retain 75∼100% of wild-type activity, suggesting that hydrophilic residues are important in hydrogen-bonding network in this position. Menard *et al.*, [15], [16] reported that Asp158 in papain is in proximity to active site His and is involved in stabilizing the thiolate-imidazolium ion pair by hydrogen-bonding interactions. However, the charge and the hydrogen bonds of Asp158 in papain both contribute to the activity of the enzyme. The molecular modeling of BntD showed that Leu76, Gly77, and Thr80 are in the close distance to Trp101 and Asp102. Replacement of Leu76 to Ala, Ser, and Val reduced colicin V activity at 37°C, but totally abolished the activity at 42°C, indicating that Leu76 is involved in interaction result in temperature-sensitivity. Site-directed mutagenesis on Gly77 to Ala and Ser showed 50% colicin V activity, but G77P lost all activity, suggesting that Gly77 residue is involved in secretion activity but is not involved in turning the structure.

Several residues surrounding critical residue Cys32 were tested and only Gln26 affected colicin V secretion activity. Sequence alignment shows that Gln26 corresponding to Gln19 in papain is conserved among cysteine proteases. Gln19 is reported to form an “oxyanion hole" with Cys25 that stabilizes the Cys-His ion pair. In colicin V system, Gln26 mutants show reduced colicin V activity.

This work identifies Asp as the third residue of catalytic triad of His-Cys-Asp, and shows that additional surrounding residues and their sequence alignments contribute to the cysteine protease activity and the secretion of active colicin V.

## Materials and Methods

### Media and growth conditions

TB (10 g of tryptone and 8 g of NaCl per liter) was used as both liquid and solid (with 1.5% agar) growth media for transformation. TB plates with 0.7% agar as semi- solid growth media for lawn were used for colicin V secretion assay [2], [30]. Ampicillin and chloramphenicol were used at final concentrations of 100 µg/ml and 30 µg/ml, respectively.

### Bacterial strains, plasmids and reagents

The strains and plasmids used in this study are listed in Table S1. *Escherichia coli* DH5α was used as the bacterial hosts. *E. coli* colicin V sensitive strain 71–18 for colicin V halo activity assay [2], [30], and colicin plasmids were obtained from Dr. R. Kolter (Harvard Medical School, Boston, MA). Plasmid pHK11-4 contains intact *cvaA* and *cvaC* but not *cvaB*. For colicin V secretion assays, *cvaB* and its mutations were provided by a complementary plasmid pACYC184. Recombinant DNA manipulations were performed essentially as described by Sambrook *et al* [31]. The expression vector pTrcHis2B with His_6_-tag at C-terminus was from Invitrogen (Carlsbad, CA).

### Site-directed mutagenesis

All mutagenesis were carried out by PCR using oligonucleotides containing the desired mutations. The standard PCR reaction in a total volume of 50 µl contained 20 ng of linearized plasmid template, 400 ng of each oligonucleotide primers, 200 µM of each deoxynucleotide triphosphate, and 5 units of Ex *Taq* DNA polymerase (PanVera/TaKaRa, Madison, WI) in the buffer provided by the manufacturer.

To generate the desired site-directed mutagenesis constructs, a three-round asymmetric PCR strategy was used as previously described [10], [32]. All mutations were confirmed by sequencing using an ABI Prism Big Dye Terminator Cycle Sequencing Ready Reaction Kit (Applied Biosystems, Foster City, CA) and appropriate primers.

### Protein sequence alignment and dynamic protein modeling

The N-terminal CvaB (BntD, amino acids 1–171) has previous determined as the protease domain [10]. The BntD protein sequence was aligned with other C39 peptidase family by using the ClustalW2 multiple sequence alignment (http://www.ebi.ac.uk/Tools/msa/clustalw2/) [33].

Coordinates of the crystal structures of the *E. coli* translation inhibitor (PDB: 1JD1) and photosynthesis metal transporter (PDB: 1G8P) were obtained from protein database (www.rcsb.org/pdb) and used as templates for the molecular modeling. The BntD sequence was aligned with the templates using BLAST (www.ncbi.nlm.nih.gov/BLAST) and FASTA (www.ebi.ac.uk/fasta33). The corresponding residues of CvaB were graphically incorporated into the published crystallographic coordinates of templates using program AMMP (http://www.cs.gsu.edu/~cscrwh), creating the structure for the dynamic modeling computations. The computations, including energy minimization and molecular dynamics, generate minimized structure, which is displayed using RasMol molecular display program (http://www.bernstein-plus-sons.com/software/rasmol).

### Colicin V activity assay

Direct colony halo assays were performed as described in Skvirsky *et al.* [30] with modifications [10]. DH5α cells containing various plasmids grown overnight in TB broth were spotted on colicin V sensitive 71-18-overlaid TB plates and incubated at 30°C, 37°C, or 42°C. All the results were shown as 37°C unless otherwise indicated. The halo area around colony was measured overnight or at different time as indicated during incubation. Amino acids are shown in the one-letter code. The description of the mutations represented the amino acid change. Letters before the number indicates the original amino acid residues, and the letter after the number indicates the introduced amino acid residues. The symbol represented colicin V secretion activity: Wild-type (++++) is used as 100% secretion activity. (++) is used as approximate 50% secretion activity compared to the wild type, + is used as 25%. Symbol “−" indicates no secretion activity.

### Protein Purification and Protease activity assay

N-terminal CvaB (BntD) was cloned, expressed, purified and used for proteolytic assay as previous described [10]. The reaction mixtures contained 4.32 µg purified BntD with protease reaction buffer containing 50 mM Tris-HCl, pH 7.6, 5 mM CaCl_2_, 10 mM 2-mercaptoethanol, and 5 mM dithiothreitol and were first incubated at 37°C for 10 mins. The substrate, L-arginine *p*-nitroanilide (LA-pNA) was added into the mixture with a final concentration of 1 mM. The cleavage product, p-nitroaniline, was monitored at various times in a Bio-Rad spectrophotometer at O.D. 405 nm. Background of hydrolysis of the substrate without BntD was subtracted.

### Membrane preparations and Western immunoblots

DH5α cells containing appropriate plasmids grown overnight in TB at the presence of appropriate antibiotics at 30°C were diluted to an OD_600_ of ∼0.1 and incubated for growth. When OD_600_ reached ∼0.5, the cells were spun down and washed once by TB, and the cells were re-suspended in TB, induced with 0.5 mM 2,2′-dipyridyl, and harvested 2 hr later. Cells were suspended in 50 mM Tris - HCl (pH 8.0) - 50 mM NaCl - 20% glycerol plus Complete Protease Inhibitor Cocktail (Roche, IN) and 1 mM *N*-ethylmaleimide (NEM), and lysed with a French Press as described [10]. Lysates were centrifuged for 8 min at 4,000×*g* to remove cell debris, and the supernatants were centrifuged at 100,000 rpm for 20 min at 4°C in a Beckman TLA100.3 rotor. The pellet fractions containing total membranes were suspended in sample buffer containing 1 mM NEM, complete protease inhibitor cocktail, and 4% SDS. For detection of CvaB and CvaA, membranes were incubated at 37°C for 30 min prior subjecting to 10% SDS-PAGE. For Western immunoblottings, alkaline phosphate-conjugated goat anti-rabbit immunoglobulin G (Bio-Rad Laboratories, Hercules, CA) was used as the secondary antibody for anti-CvaA [34], anti-N-terminal CvaB and anti-colicin V antibody [10]; alkaline phosphate-conjugated goat anti-mouse immunoglobulin G (Promega, Madison, WI) was used as the secondary antibodies for anti-Flag antibodies which was obtained from Sigma (St. Louis, MO).

## Supporting Information

Table S1 **Bacterial strains and plasmids.** The strains and plasmids used in this study are listed. The details are defined as in Material and Methods.
(DOCX)
