# Supplementary material for: Mutational Analysis of Cvab, an ABC Transporter Involved in the Secretion of Active Colicin V
Source: PLoS One. 2012 Apr 23;7(4):e35382. doi: 10.1371/journal.pone.0035382 (PMC3335142; doi:10.1371/journal.pone.0035382)
Supplement: Table S1 — Bacterial strains and plasmids. The strains and plasmids used in this study are listed. The details are defined as in Material and Methods. (DOCX) [file pone.0035382.s001.docx]

SI Table1. Bacterial strains and plasmids

| **Strain and Plasmids** | **Genotype or Description** | **Reference** |
| --- | --- | --- |
| Strain DH5α | *supE*44 Δ*lac*U169(*φ*80 *lacZ*ΔM15) *hsd*R17 *rec*A1 *end*A1 *gyr*A96 *thi*-1 *rel*A1 | Hanahad 1983 |
| Strain 71-18 | *supE* *thi* Δ(*lac*-*proAB*) F′ [*proAB*+ *lacI*q *lac*ZΔM15] | Yanisch *et al*., 1985 |
|  |  |  |
| **Plasmids** |  |  |
| pHK11-4 | pHK11 with c*va*B:: Tn5 | Gilson *et al*., 1987 |
| pKW67 | pACYC184 with wild-type CvaB 698 amino acids | This Study |
| pKW182 | Q26N from pKW67 |  |
| pKW183 | Q26K from pKW67 |  |
| pKW184 | Q26E from pKW67 |  |
| pKW364 | Q26H from pKW67 |  |
| pKW177 | N70K from pKW67 |  |
| pKW380 | N70G from pKW67 |  |
| pKW185 | N115G from pKW67 |  |
| pKW186 | N115K from pKW67 |  |
| pKW187 | W101A from pKW67 |  |
| pKW235 | W101K from pKW67 |  |
| pKW239 | W101S from pKW67 |  |
| pKW232 | W101D from pKW67 |  |
| pKW233 | W101F from pKW67 |  |
| pKW244 | W101Y from pKW67 |  |
| pKW240 | W101P from pKW67 |  |
| pKW250 | W101H from pKW67 |  |
| pKW236 | D102A from pKW67 |  |
| pKW245 | D102E from pKW67 |  |
| pKW234 | D102S from pKW67 |  |
| pKW241 | D102K from pKW67 |  |
| pKW269 | D102N from pKW67 |  |
| pKW270 | D102Q from pKW67 |  |
| pKW138 | D102C from pKW67 |  |
| pKW277 | D102W from pKW67 |  |
| pKW280 | D102F from pKW67 |  |
| pKW281 | D102P from pKW67 |  |
| pKW287 | D102G from pKW67 |  |
| pKW288 | D102H from pKW67 |  |
| pKW289 | D102Y from pKW67 |  |
| pKW279 | W101D&D102W from pKW67 |  |
| pKW238 | V108A from pKW67 |  |
| pKW237 | V108S from pKW67 |  |
| pKW243 | V108K from pKW67 |  |
| pKW242 | V108D from pKW67 |  |
| pKW248 | V108L from pKW67 |  |
| pKW212 | Wild type CvaB with epitope flag at C-terminus |  |
| pKW214 | V108A CvaB with epitope flag at C-terminus |  |
| pKW421 | W101Y CvaB with epitope flag at C-terminus |  |
| pKW422 | W101K CvaB with epitope flag at C-terminus |  |
| pKW423 | W101P CvaB with epitope flag at C-terminus |  |
| pKW424 | W101H CvaB with epitope flag at C-terminus |  |
| pKW306 | D120K CvaB with epitope flag at C-terminus |  |
| pKW301 | D102F CvaB with epitopeflag at C-terminus |  |
| pKW303 | D102A CvaB with epitope flag at C-terminus |  |
| pKW302 | D102P CvaB with epitope flag at C-terminus |  |
| pKW304 | D102W&W101D CvaB with epitope flag at C-terminus |  |
| pYH53 | D121A from pKW67 |  |
| pYH59 | D121S from pKW67 |  |
| pYH61 | D121N from pKW67 |  |
| pYH55 | P122A from pKW 67 |  |
| pYH63 | P122S from pKW67 |  |
| pYH65 | P122G from pKW67 |  |
| pYH68 | D121E from pKW67 |  |
| pYH70 | D122V from pKW67 |  |
| pYH72 | D122F from pKW67 |  |
| pYH74 | D122Y from pKW67 |  |
| pYH84 | D121P from pKW67 |  |
| pYH86 | P122D from pKW67 |  |
| pYH88 | P122E from pKW67 |  |
| pYH90 | P122N from pKW67 |  |
| pYH92 | D121P&P122D from pKW67 |  |
